# X-ray crystallographic and kinetic studies of biguanide containing aryl sulfonamides as carbonic anhydrase inhibitors†

**DOI:** 10.1039/d4md01018c

**Published:** 2025-01-24

**Authors:** Chiara Baroni, Murat Bozdag, Gioele Renzi, Viviana De Luca, Clemente Capasso, Carla Bazzicalupi, Silvia Selleri, Marta Ferraroni, Fabrizio Carta, Claudiu T. Supuran

**Affiliations:** a Department of Chemistry “Ugo Schiff”, University of Florence Via della Lastruccia 3 50019 Sesto Fiorentino FI Italy marta.ferraroni@unifi.it; b NEUROFARBA Department, University of Florence Via Ugo Schiff 6 50019 Sesto Fiorentino FI Italy fabrizio.carta@unifi.it; c Department of Biology, Institute of Bioscience and Bioresources (IBBR)-CNR Via P. Castellino 111 80131 Napoli NA Italy

## Abstract

Here, we report a small series of dual-targeting compounds that combine the prototypical carbonic anhydrase (CA) zinc-binding sulfonamide moiety with the biguanide group of metformin, an emerging anticancer drug. The compounds reported similar *in vitro* inhibition profiles on a panel of physiologically relevant human (h)CAs, with marked selectivity for the cancer related IX and XII isoforms. The binding modes of representative inhibitors 5b and 5c within the active site of the hCA isoforms II and XII-mimic were assessed by X-ray crystallography, thus allowing us to clarify molecular features that may be useful for the design of more specific and potent inhibitors. For instance, we identified a mutation in the hCA XII-mimic which was found responsible for the selectivity of the ligands toward the tumor associated isoform. Interestingly, in the hCA II/5c complex, a second inhibitor molecule was bound to the catalytic cleft, probably affecting the inhibition properties of the canonical zinc-bound inhibitor.

## Abstract 2

Dual-targeting carbonic anhydrase (CA) inhibitors bearing the biguanide group of metformin showed good selectivity against the cancer related CA IX and XII. The X-ray structures of two selected inhibitor complexes with hCA II and XII were solved.

## Graphical abstract

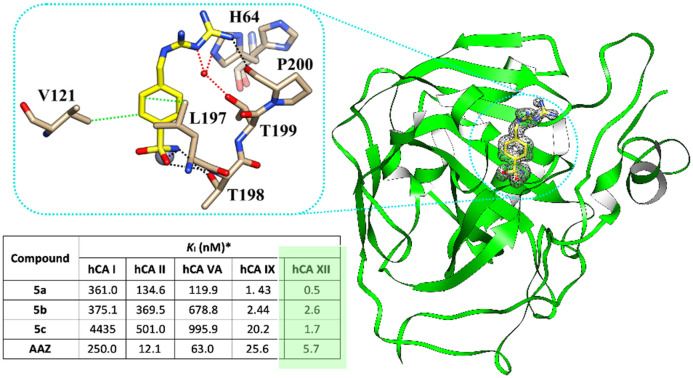

## Introduction

Carbonic anhydrases (CAs) are a family of zinc metalloenzymes that catalyze the reversible hydration of carbon dioxide to bicarbonate ions and protons.^1^ Twelve catalytically active CA isoforms are expressed in humans, showing different localization patterns.^2^ Among the human (h)CAs, the membrane-bound isoforms IX and XII are recognized as significant solid tumor markers being overexpressed in various cancer cell lines mainly induced by hypoxia regulating factors (*i.e.* HIFs), and in the case of XII also by additional effectors.^3,4^ The crucial role of CA IX/XII in cancer cells is maintaining the intracellular pH at ∼6.5 to grant cells survival after the Warburg effect is established.^5,6^ Since such isoforms are constitutively expressed only in gut tissues, they assume considerable importance as anticancer targets.^7^

The onset and progression of tumor growth involve numerous aberrant proteins and signaling pathways, highlighting the complexity of developing effective therapies. In contrast to single-targeting drugs traditionally used in association with each other, the development of chemical entities endowed with multi-pharmacological features provides clear advantages, such as improved patient compliance and more predictable pharmacokinetics.^8^

Hence, in the last decade, multitarget-based approaches have also been exploited based on high-affinity CA IX and XII inhibitors of the sulfonamide, coumarin, and sulfocoumarin types.^9–13^ Original approaches included the epidermal growth factor receptor (EGFR),^14^ telomerase enzyme,^15^ and the P-glycoprotein transporter as additional targets.^16^

Metformin is a widely used antidiabetic drug.^17^ In addition to its role in glycemic control in type 2 diabetes, it has been recently identified with cardiovascular protection, anti-inflammatory and anticancer properties.^18^ The anticancer effects of metformin are attributed to both direct and indirect actions. Indirectly, metformin reduces gluconeogenesis, decreases hyperinsulinemia, and mediates the adenosine monophosphate protein kinase (AMPK) activation. All these pathways are believed to contribute to reducing cancer cell growth and progression.^19–21^ In addition, metformin directly suppresses mitochondrial activity and reduces the availability of adenosine triphosphate (ATP) and, more in general, those biosynthetic precursors required for cell growth.^22–24^ Valuable studies indicated that metformin acted as an inhibitor of the mammalian target of rapamycin (mTOR) signaling^25–28^ and modulates the PD-L1/PD-1 axis *via* AMPK-dependent and independent mechanisms.^18,29,30^

Here, we report a small series of compounds designed to merge within a single chemical scaffold, the prototypic CA inhibitor (CAI) moiety (*i.e.* the primary sulfonamide), and the biguanide group of metformin. The compounds obtained were investigated *in vitro* for their ability to inhibit the most relevant hCAs and their binding mode in the complex with hCAs II and XII-mimic was assessed using X-ray crystallographic studies.

## Results and discussion

### Synthesis of inhibitors of the carbonic anhydrases

**Scheme 1:**
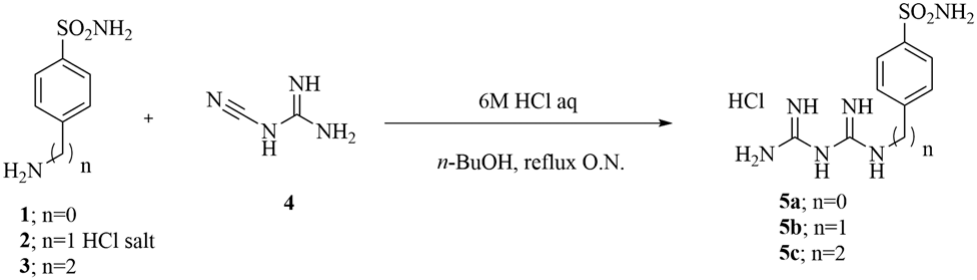
Synthetic procedure for compounds 5a–c.

The final compounds 5a–c were synthesized by adding commercially available amines, bearing the primary sulfonamide moiety, to cyanoguanidine in *n*-butanol using a stoichiometric amount of hydrochloric acid solution in water (Scheme 1).^19^

Synthesized compounds were designed to carry a positive charge, ensuring their impermeability to membranes. The inclusion of a bis-guanidine moiety induces highly polar features in these compounds, allowing potential double protonation at a physiological pH (at the two guanidine moieties). The obtained compounds were purified through crystallization from isopropyl alcohol (IPA) and were fully characterized using ^1^H-NMR, ^13^C-NMR, and mass spectrometry (ESI-MS).

### Enzyme inhibition studies

Compounds 5a–c were evaluated *in vitro* for their ability to inhibit the physiologically relevant CA isoforms I, II, VA, IX, and XII through the stopped-flow technique.^31^ Kinetic effectiveness was expressed as the means of the K_I_ values in Table 1 and it was compared to the standard CAI acetazolamide (AAZ).

**Table 1:** Inhibition of cytosolic hCAs I and II, mitochondrial VA, membrane-associated IX, and XII by a CO_2_ hydrase stopped-flow assay using acetazolamide (AAZ) as a reference drug^31^

Compound	*K*_I_ a (nM)				
	hCA I	hCA II	hCA VA	hCA IX	hCA XII
5a	361.0	134.6	119.9	1.43	0.5
5b	375.1	369.5	678.8	2.44	2.6
5c	4435	501.0	995.9	20.2	1.7
AAZ	250.0	12.1	63.0	25.6	5.7

a Mean from 3 different assays, by the stopped-flow technique (errors were in the range of ±5–10% of the reported values).

The above reported compounds showed low inhibitory activity towards the cytosolic isoforms hCA I and II and mitochondrial VA with *K*_I_ values spanning between high and low-micromolar values (*K*_I_ values of 134.6 and 4435 nM). Overall, the structure–activity relationship (SAR) determined for these compounds indicates a progressive reduction of the inhibition potencies with the increase in the alkyl spacer length connecting the biguanide with the aryl sulfonamide groups (Table 1).

As for the tumor associated CAs IX and XII, all compounds showed high inhibition potencies. For instance, the hCA IX associated *K*_I_ values ranging between 1.43 and 20.2 nM was lower than that of the reference AAZ (*i.e. K*_I_ = 25.6 nM). Elongation of the tether in 5a to afford 5b reduced the inhibition potency up to 1.7-fold (*K*_I_ values of 1.43 and 2.44 nM for 5a and 5b, respectively). Further elongation, as in 5c, significantly enhanced the *K*_I_ value up to 8.3-fold and thus was comparable to the reference AAZ (*i.e. K*_I_ of 20.2 nM).

All compounds showed remarkable selectivity for hCA XII as they exhibited *K*_I_ values between 0.5 and 2.6 nM, and thus up to 11.4-fold lower than that of AAZ (*i.e. K*_I_ = 5.7 nM). In this case, the progressive elongation of the alkyl tether did not induce a dependent reduction of the ligand affinity as demonstrated for the derivative 5c, which was 1.5-fold more effective than its shorter derivative 5b (*i.e. K*_I_ values of 2.6 and 1.7 nM for 5b and 5c respectively).

Overall, the *in vitro* data in Table 1 referring to 5a, 5b and 5c showed marked potency and high selectivity for the tumor associated hCAs IX and XII over the cytosolic and mitochondrial isoforms. Such a kinetic fingerprint makes these compounds the ideal candidates for the development of CAI-based multitargeting new generation anticancer drugs.

### Crystallographic studies

**Fig. 1:**
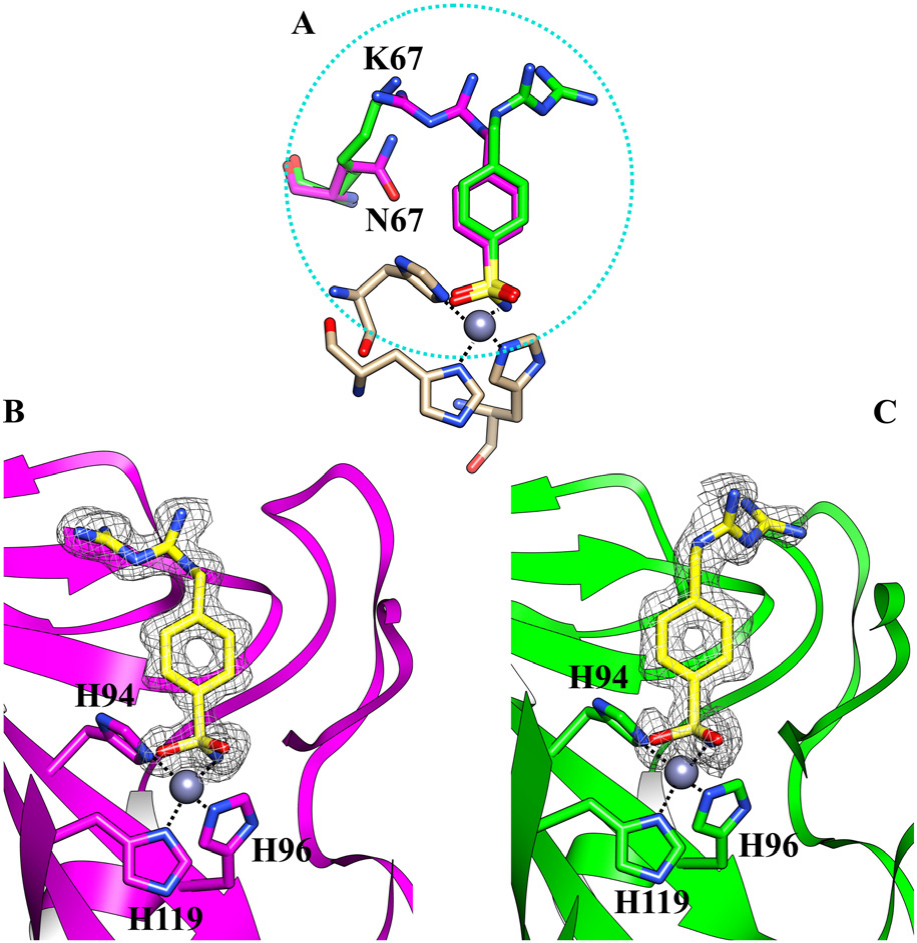
(A) Comparison of the two different orientations of the inhibitor 5b within the active site of hCA II (magenta) and hCAXII-mimic (green), respectively; (B) the active site of hCA II complexed with inhibitor 5b (PDB: 8ROU); (C) the active site of hCAXII-mimic complexed with inhibitor 5b (PDB: 8RNS). The 2Fo–Fc density map for the inhibitor is also shown.

We determined the X-ray crystallographic structures of hCA II and hCA XII-mimic in the complex with inhibitor 5b (Fig. 1). CA XII-mimic is an engineered CA II with a modified sequence constructed to mimic the CAXII active site.^32,33^ The structures were solved using the data collected on flash-frozen crystals with PEG 400 as a cryoprotectant (see the Experimental part and discussion below).

Analysis of the electron density maps revealed a clear density for the benzenesulfonamide group of the inhibitor, confirming its binding to the enzymes.

**Fig. 2:**
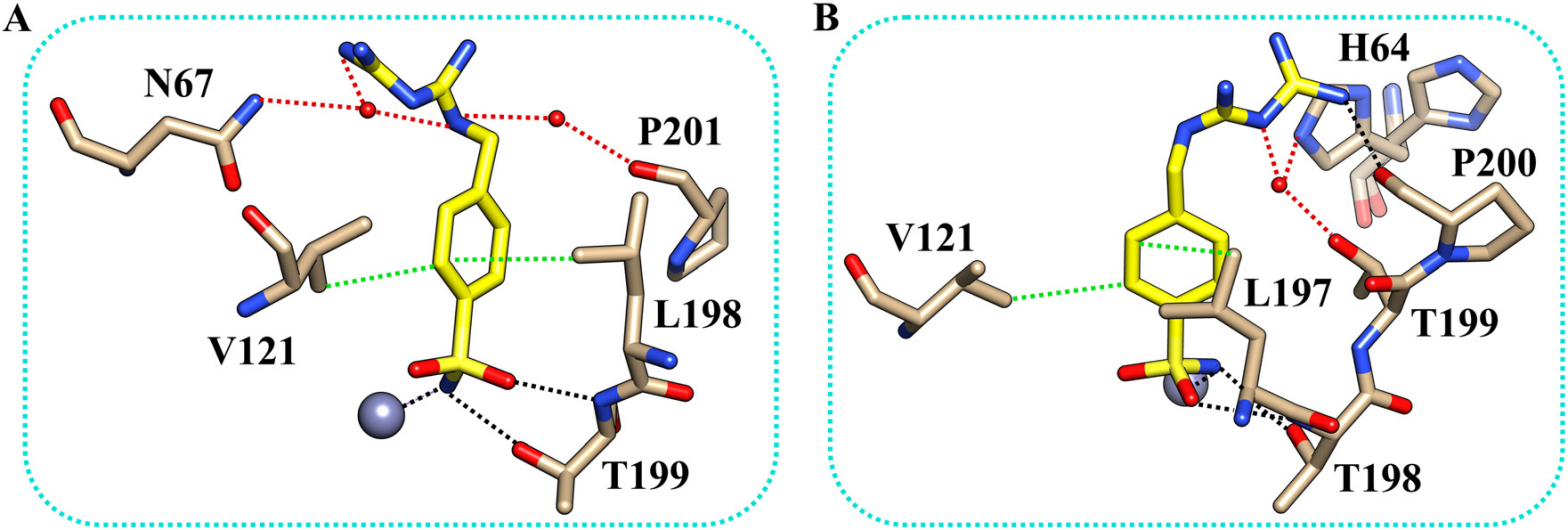
The binding mode of inhibitor 5b within the active site of hCA II (A) and hCA XII-mimic (B). Residues involved in the binding of the inhibitor are also shown. Hydrogen bonds, water bridges, and hydrophobic interactions are depicted as black, red, and green dotted lines. The gray sphere represents the zinc ion in the protein active site. The side chain of His64 was modeled in two conformations.

The inhibitor presents the classical binding mode of the sulfonamide moiety in the active site of the two isoforms by directly interacting with the zinc ion and forming hydrogen bonds with Thr199/198 (numbering of hCA II and hCA XII, respectively) (Fig. 2). On the other hand, different hydrogen bonds and water bridge interactions are established within the active site of the two enzymes, resulting in two different orientations of the inhibitor tail. In fact, in the hCA II–5b complex, the nitrogen atoms of the biguanide group form water bridges with Asn67. In contrast, in the hCAXII–5b complex, the inhibitor interacts with the amino acid residues in the opposite region of the protein cavity. Indeed, the nitrogen atoms of the biguanide group establish water bridges with Thr199 and His64. The phenyl ring of the inhibitor also has hydrophobic interactions with Val121 and Leu197/198.

By analyzing the mutations in the active site of hCAXII-mimic with respect to the hCA II isoform, we realized that the Asn67Lys variation in the hCA XII-mimic amino acid sequence is the only one that could influence the binding mode of the inhibitor. Lysine is more flexible and bulkier than asparagine, which may induce a different organization of the inhibitor within the enzyme cavity due to steric hindrance (Fig. 1A). The inhibitor tail in hCA II forms a water bridge with Asn67, whereas in hCA XII-mimic it is oriented in the opposite direction of Lys67.

**Fig. 3:**
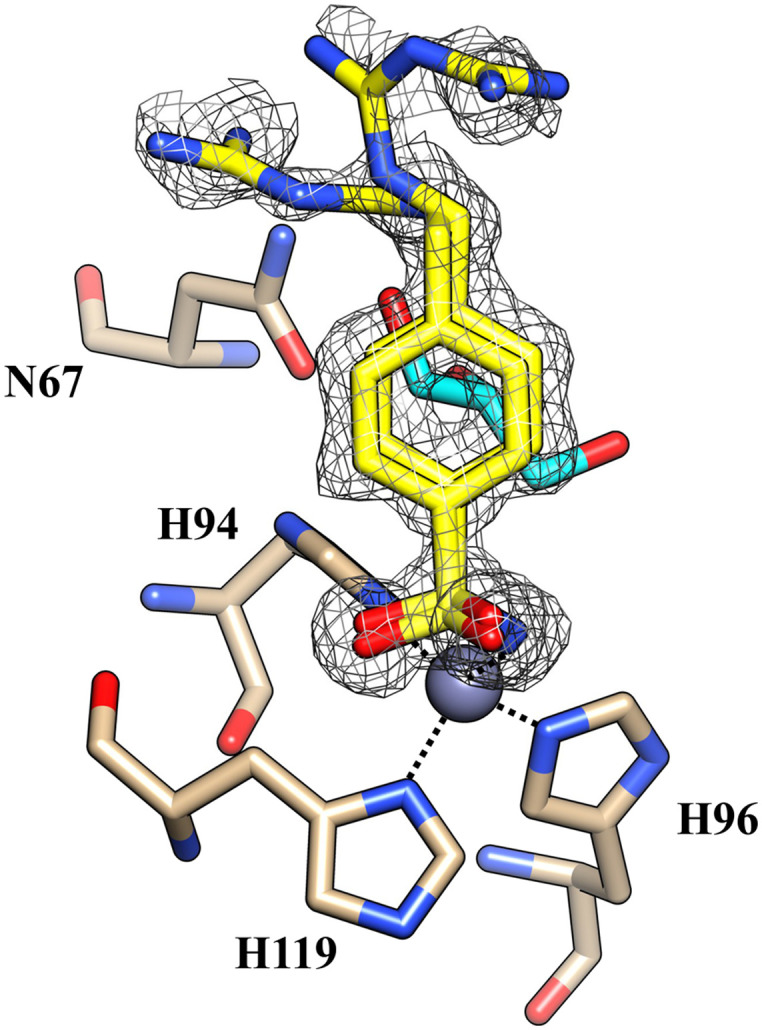
The active site of hCA II complexed with inhibitor 5b (PDB: 8ROW) from data collected using glycerol as a cryoprotectant. Glycerol is depicted in cyan.

Furthermore, no cryoprotectant molecules were detected within the active site of the isoforms, in contrast to what was observed in previous crystallographic analysis performed using glycerol as a cryoprotectant (Fig. 3).

Glycerol is the most widely used cryoprotecting agent in X-ray protein crystallography.^34^ Hence, it is routinely used to prevent ice formation in freezing CA crystals of different isoforms and their complexes with inhibitors. Often, glycerol penetrates the protein active site, establishing hydrogen bonds with surrounding amino acid residues, which may alter the inhibitor binding mode. Indeed, our study found that glycerol is bound in the active site of hCA II in the complex with 5b. Moreover, the inhibitor is disordered and its tail was modeled in two conformations, with different occupational values, as shown in Fig. 3. The disorder may be due to the steric hindrance of the cryoprotectant molecule which influences the complex formation. To avoid the presence of the cryoprotectant molecule within the active site, the analyses were repeated using a higher molecular dimension cryoprotectant agent, PEG 400. In this case, as already shown, we found that differently, the inhibitor assumes a unique orientation in the protein cavity (Fig. 1B), representing a more accurate determination of its binding mode.

**Fig. 4:**
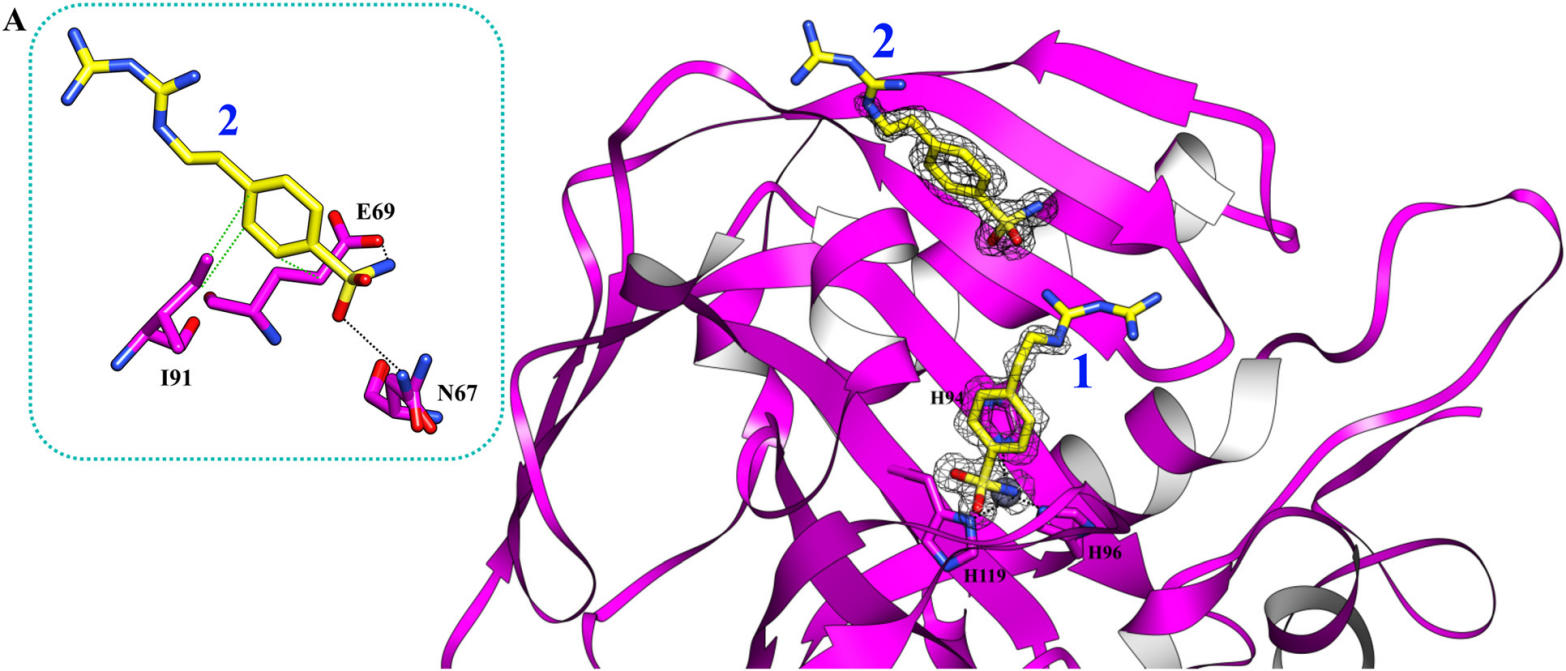
The active site of hCA II complexed with inhibitor 5c (PDB: 9H0V). Molecules of the inhibitor are labeled as 1 and 2. The binding mode of molecule 2 is shown in panel A. Hydrogen bonds and hydrophobic interactions are depicted as black and green dotted lines.

Using PEG 400 as a cryoprotectant, we also determined the structure of hCA II and hCA XII-mimic in the complex with the inhibitor 5c. The density maps of the hCA XII-mimic complex with inhibitor 5c do not show the biguanide portion of the molecule, preventing an accurate determination of its binding mode (data not shown). A similar situation is observed for the hCA II complex, where the biguanide group position is also unclear (Fig. 4). However, it is noteworthy that two distinct inhibitor molecules are present within the active site of hCA II.

In numerous complexes of hCA II with inhibitors, it has been found that a second inhibitor molecule binds near the N-terminal end of the protein (for example see ref. 35 and 36).

Nevertheless, in this case, a second inhibitor molecule is bound within the active site probably reducing the inhibitor affinity by introducing disorder within the binding cavity. This effect may explain the lower inhibition constant, *K*_I_ = 501.0 nM, in contrast to the significantly stronger affinity observed in the hCA XII–5c complex, where *K*_I_ = 1.7 nM. In panel A of Fig. 4, the binding mode of the second inhibitor molecule is shown. One oxygen and the nitrogen of the sulfonamide group establish hydrogen bonds with Asn67 and Glu69, respectively. Moreover, the phenyl ring forms hydrophobic interactions with Ile91 and Glu69. The binding of a second inhibitor molecule was not observed in the hCA XII-mimic complex and can be explained by comparing the sequences of the two isoforms. In fact, residues interacting with the inhibitor molecule 2 are mutated in the hCA XII-mimic sequence (Asn67Lys, Glu69Asn, and Ile91Thr) in a way that prevents the interaction with this second molecule bound to the upper part of the catalytic cavity.

## Experimental section

### Chemistry

Solvents and all reagents were purchased from Sigma-Aldrich, VWR, and TCI. Nuclear magnetic resonance (^1^H NMR and ^13^C NMR) spectra were recorded using a Bruker Advance III 400 MHz spectrometer in DMSO-d_6_. Chemical shifts are reported in parts per million (ppm) and the coupling constants (*J*) are expressed in hertz (Hz). Splitting patterns are designated as follows: s, singlet; d, doublet; t, triplet; m, multiplet; brs, broad singlet; dd, doublet of doublets. The assignment of exchangeable protons (NH) was confirmed by the addition of D_2_O. Analytical thin-layer chromatography (TLC) was carried out on Merck silica gel F-254 plates. Flash chromatography purifications were performed on Merck silica gel 60 (230–400 mesh ASTM) as the stationary phase, and ethyl acetate, *n*-hexane, acetonitrile, and methanol were used as eluents. The solvents used in MS measurements were acetone, acetonitrile (Chromasolv grade) purchased from Sigma-Aldrich (Milan, Italy) and mQ water 18 MΩ, obtained from Millipore's Simplicity system (Milan, Italy). The mass spectra were obtained using a Varian 1200 L triple quadrupole system (Palo Alto, CA, USA) equipped with an electrospray source (ESI) and negative ions. Stock solutions of analytes were prepared in acetone at 1.0 mg mL^−1^ and stored at 4 °C. Working solutions of each analyte were freshly prepared by diluting stock solutions in a mixture of mQ H_2_O/ACN 1/1 (v/v) up to a concentration of 1.0 μg mL^−1^. The mass spectra of each analyte were acquired by introducing, *via* a syringe pump at 10 L min^−1^, the working solution. Raw data were collected and processed with Varian Workstation, version 6.8, software. All compounds reported here are >96% purity by NMR.

### General synthetic procedure for compounds 5a, 5b and 5c

The synthesis of compounds 5a–c was carried out by following the previously reported experimental procedure.^37^ Specifically, the appropriate arylsulfonamide derivatives 1–3 (0.5 g, 1.0 equiv.) reacted with cyanoguanidine 4 (1.0 equiv.) in *n*-butanol (5 mL) followed by the addition of 6 M aqueous HCl (1.0 equiv.). The reaction mixture was heated to 100 °C under stirring for 6 h, and then the solvents were removed under vacuum to afford a residue that was crystallized from isopropyl alcohol (IPA) to obtain the desired products as white solids.

#### 4-(3-Carbamimidoylguanidino)benzenesulfonamide hydrochloride salt 5a

Yield: 40%. ^1^H-NMR (400 MHz, DMSO-d_6_) *δ* 9.91 (brs, 1H, exchangeable with D_2_O), 7.72 (d, *J* = 8.9 Hz, 2H), 7.59–7.41 (m, 6H, 4H exchangeable with D2O), 7.26 (s, 2H, exchangeable with D_2_O), 7.09 (s, 2H, exchangeable with D_2_O). 13C-NMR (100 MHz, DMSO-d_6_) *δ* 162.5, 155.3, 143.1, 138.8, 127.4, 120.5. Experimental data are in agreement with the reported literature.^38^

#### 4-((3-Carbamimidoylguanidino)methyl)benzenesulfonamide hydrochloride salt 5b

Yield: 58%. ^1^H-NMR (400 MHz, DMSO-d_6_) *δ* 7.72 (d, *J* = 8.3 Hz, 2H), 7.52 (d, *J* = 8.3 Hz, 2H), 7.41 (brs, 4H, exchangeable with D_2_O), 7.25 (s, 2H, exchangeable with D_2_O), 7.08 (brs, 2H, exchangeable with D_2_O), 3.91 (m, 2H). ^13^C NMR (100 MHz, DMSO-d_6_) *δ* 163.9, 143.6, 142.5, 130.1, 126.9, 119.7, 46.8.

#### 4-(2-(3-Carbamimidoylguanidino)ethyl)benzenesulfonamide hydrochloride salt 5c

Yield: 50%. ^1^H-NMR (400 MHz, DMSO-d_6_) *δ* 8.03 (brs, 3H, exchangeable with D_2_O), 7.78 (d, *J* = 8.3 Hz, 2H), 7.46 (d, *J* = 8.3 Hz, 2H), 7.35 (s, 2H, exchangeable with D_2_O), 3.06 (m, 2H), 2.97 (m, 2H). ^13^C-NMR (100 MHz, DMSO-d_6_) *δ* 163.9, 143.6, 142.5, 133.8, 130.1, 126.9, 36.5, 33.6. Experimental data are in agreement with the reported literature.^19^

### Carbonic anhydrase inhibition

An Applied Photophysics stopped-flow instrument was used to evaluate the ability of the test compounds to inhibit the CA-catalyzed CO_2_ hydration. Phenol red (at a concentration of 0.2 mM) was used as an indicator, working at a maximum absorbance of 557 nm, with 20 mM HEPES (pH 7.4) as a buffer, and 20 mM Na_2_SO_4_ (to maintain constant ionic strength), following the initial rates of the CA-catalyzed CO_2_ hydration reaction for a period of 10–100 s. The CO_2_ concentrations ranged from 1.7 to 17 mM for the assessment of the kinetic parameters and inhibition constants. Enzyme concentrations varied between 5 and 12 nM. For each inhibitor, at least six traces of the initial 5–10% of the reaction were used to determine the initial velocity. The uncatalyzed rates were calculated in the same manner and subtracted from the total observed rates. Stock solutions of each inhibitor (0.1 mM) were prepared in distilled–deionized H_2_O and dilutions up to 0.01 nM were done thereafter with the assay buffer. Inhibitor and enzyme solutions were preincubated together for 15 min at r.t. before the assay, to allow the formation of the E–I complex. The inhibition constants were obtained by nonlinear least-squares methods using PRISM 3 and the Cheng–Prusoff equation as reported earlier^39^ and represent the mean from at least three different determinations. hCAs I and II were purchased from Merck, while other isoenzymes were obtained in-house.^40^

### Expression and purification of the hCA XII mimic

To generate the hCA XII mimic,^33^ the CA II gene was used as the template, and nine specific mutations were introduced to reproduce key features of the hCA XII active site. These mutations were directly incorporated into the gene sequence during its design. The modified gene, containing these mutations, was synthesized by Genescript and subcloned into the pET43 vector using BamHI and XhoI restriction sites. Competent BL21 (DE3) cells were transformed with the plasmid containing the engineered gene. A fresh culture was grown at a 1 L scale in an LB broth supplemented with 100 μg mL^−1^ ampicillin. The cells were incubated at 37 °C until reaching the mid-log phase (OD_600_ = 0.6–0.7), at which point protein expression was induced by adding 0.5 mM isopropyl β-d-1-thiogalactopyranoside (IPTG). After 30 minutes, 0.5 mM ZnSO_4_ was added to the culture, which was then incubated for an additional 4.5 hours. Cells were harvested by centrifugation at 5000 rpm for 20 minutes. The pellet was resuspended in 20 mM Tris-HCl buffer (pH 8.3) and subjected to sonication with 10 cycles of 10 seconds “on” and 120 seconds “off” at 50% amplitude (Bandelin Sonoplus). The lysate was clarified by centrifugation at 12 000 × *g* for 45 minutes. The supernatant was incubated with nickel affinity gel resin (His Select HF, Sigma), and pre-equilibrated in lysis buffer for 30 minutes. The resin was separated by centrifugation at 1500 × *g* and washed with a buffer containing 50 mM Tris-HCl (pH 8.3), 500 mM NaCl, and 20 mM imidazole. The protein was eluted using the same buffer supplemented with 250 mM imidazole. Eluted fractions were dialyzed against 20 mM Tris-HCl (pH 8.3). The purified recombinant protein was confirmed to be 95% pure, with a yield of approximately 3.2 mg per liter of bacterial culture. The engineered variant retained catalytic activity, demonstrating that the nine introduced mutations in the binding site did not disrupt the enzyme's intrinsic catalytic mechanism.

### Crystallography

#### Crystallization and X-ray data collection

Crystals of the hCA XII-mimic isoform (protein solution 8 mg mL^−1^ in Tris-HCl 20 mM, pH 8.3) were grown *via* the hanging drop vapor diffusion method in a 24-well Linbro plate. Drops were prepared using 1 μL protein solution mixed with 1 μL reservoir solution containing 1 M sodium citrate and 50 mM Tris-HCl (pH 7.5) and were equilibrated against 500 μL of the same solution at 296 K. Crystal growth was observed after fifteen days.

hCA II purchased from Sigma was dissolved in 50 mM Tris-HCl (pH 8.0) to obtain a final concentration of 10 mg mL^−1^.

Crystals of the hCA II isoform were obtained using the hanging drop vapor diffusion method in a 24-well Linbro plate. One microliter of hCA II was mixed with 1 μL of a solution of 1.5 M sodium citrate and 50 mM Tris-HCl (pH 8.0) and equilibrated against 500 μL of the same solution at 296 K. Crystal growth was observed after five days.

The complexes of the two isoforms with the inhibitors 5b and 5c were prepared by soaking the crystals in the mother liquor solution containing the inhibitor at a concentration of 10 mM for 2 hours.

Initially, all crystals were flash-frozen at 100 K using a solution obtained by adding 20% (v/v) glycerol to the mother liquor solution as a cryoprotectant. However, from the data obtained, we found that cryoprotectant molecules interact with the inhibitor within the protein active site. Hence, to avoid the cryoprotectant presence inside the enzyme active site with possible alteration of the inhibitor binding mode, we collected data using a different cryoprotectant solution obtained by adding 15% (v/v) of PEG 400 to the mother liquor solution. X-ray diffraction data were collected using synchrotron radiation at the XRD2 beamline at Elettra Synchrotron (Trieste, Italy) with a DECTRIS Pilatus 6 M detector.

#### Structure determination

The crystal structures of CA XII-mimic (PDB accession code: 4Q08) and CA II (PDB accession code: 3HS4) without solvent molecules and other heteroatoms were used to obtain the initial phases of the structures using Refmac5^41^ from the CCP4 package.^42^ Coordinates of the inhibitors were obtained using the program JLigand^43^ and introduced in the model. Five percent of the unique reflections were selected randomly and excluded from the refinement data set for Rfree calculations. The initial |*F*_o_–*F*_c_| difference electron density maps unambiguously showed the inhibitor molecules. The inhibitor 5b was introduced in the model with 1.0 occupancy. Refinements proceeded using standard protocols of positional and anisotropic atomic displacement parameters alternating with the manual building of the models using COOT.^44^ Inhibitor–enzyme interactions were analyzed using the PLIP web tool.^45^ Atomic coordinates were deposited in the PDB (hCA II-5b accession code: 8ROU; hCA XII-5b accession code: 8RNS; hCA II-5b with glycerol as a cryoprotectant: 8ROW; hCA II-5c: 9H0V). Graphical representations were generated with Chimera.^46^

## Conclusions

Herein, we reported aryl sulfonamides tailed with a biguanide moiety 5a–c as selective inhibitors of the tumor associated hCA IX and XII isoforms, and we assessed the binding modes of 5b and 5c in the complex with hCA II and XII-mimic isoforms using X-ray crystallography. For instance, the aryl sulfonamide portion of 5b was firmly placed deep into the catalytic cavities of hCAs II/XII, and the flexible biguanide tail was engaged in multiple interactions with the amino acids exposed at the top-end of the clefts, thus determining the specific orientation of the ligand as well its stabilization depending on the isoform. Noteworthily, the Asn67Lys mutation in the hCA XII-mimic was found to be mainly responsible for driving 5b toward the tumor associated isoform over the cytosolic hCA II. Our investigations allowed us to identify a second 5c ligand located at the N-terminal of the hCA II, proximal to the catalytic cleft, and thus reasonably able to influence the binding affinity of the main inhibitor. Such a mechanism may support a long-range allosteric hCA regulation mechanism not yet explored and may be potentially exploitable for the fine tuning of the metalloenzymes.

## Data availability

The data supporting this article have been included as part of the ESI.† Crystallographic data for the structures of the complexes have been deposited at the PBD under accession codes 8ROU, 8RNS, 8ROW and 9H0V.

## Conflicts of interest

There are no conflicts to declare.

## Supplementary Material

MD-016-D4MD01018C-s001
