# Supplementary material for: X-ray crystallographic and kinetic studies of biguanide containing aryl sulfonamides as carbonic anhydrase inhibitors
Source: RSC Med Chem. 2025 Jan 24;16(4):1633–40. doi: 10.1039/d4md01018c (PMC11809658; doi:10.1039/d4md01018c)
Supplement: MD-016-D4MD01018C-s001 [file MD-016-D4MD01018C-s001.pdf]

Supplementary Information (SI) for RSC Medicinal Chemistry.
This journal is © The Royal Society of Chemistry 2025

Supplementary Material for

X-ray crystallographic and kinetic studies of biguanide containing aryl sulfonamides as Carbonic anhydrase inhibitors

Chiara Baroni[1], Murat Bozdag[2], Gioele Renzi[2], Viviana De Luca[3], Clemente Capasso[3], Carla Bazzicalupi[1], Silvia Selleri[2], Marta Ferraroni[1], Fabrizio Carta[2], and Claudiu T. Supuran[2]

[1] Department of Chemistry "Ugo Schiff", University of Florence, Via della Lastruccia 3, 50019 Sesto Fiorentino (FI), Italy;

[2] NEUROFARBA Department, University of Florence, Via Ugo Schiff 6, 50019 Sesto Fiorentino (FI), Italy

[3] Department of Biology, Institute of Bioscience and Bioresources (IBBR)-CNR, via P. Castellino 111, 80131 Napoli (NA), Italy

*Corresponding author: (fabrizio.carta@unifi.it and marta.ferraroni@unifi.it)

Index

^{1}H, ^{13}C, Spectra of compounds S2-S4

Table S1. Summary of Data Collection and Refinement Statistics of hCA II-**5b**, hCAII-**5b** with glycerol and mimic-hCA XII-**5b** S5

Table S2. Summary of Data Collection and Refinement Statistics of hCA II-**5c** S6

Figure S1. The amino acid sequence of the hCA XII-mimic protein S7

^{1}H NMR spectrum of compound **5a** (400 MHz, DMSO-*d6*)

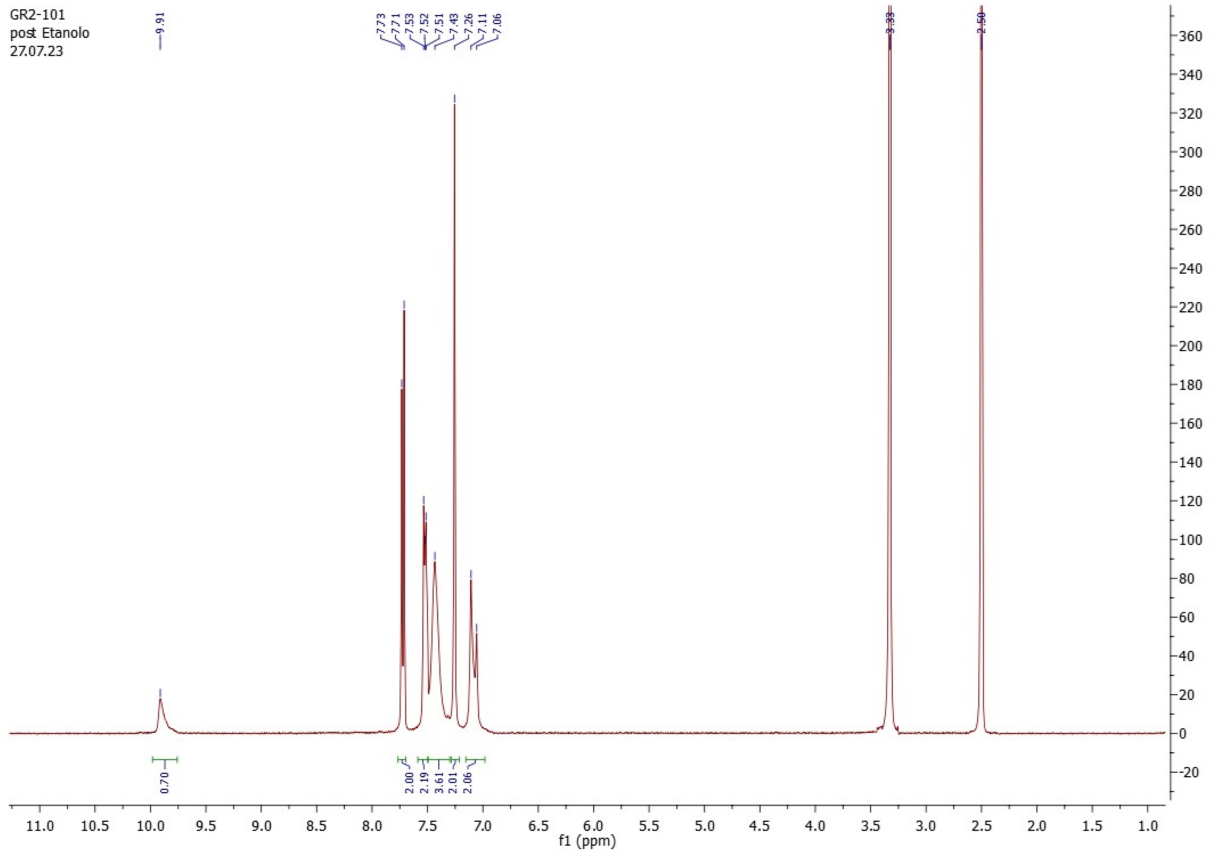

^{13}C NMR spectrum of compound **5a** (100 MHz, DMSO-*d6*)

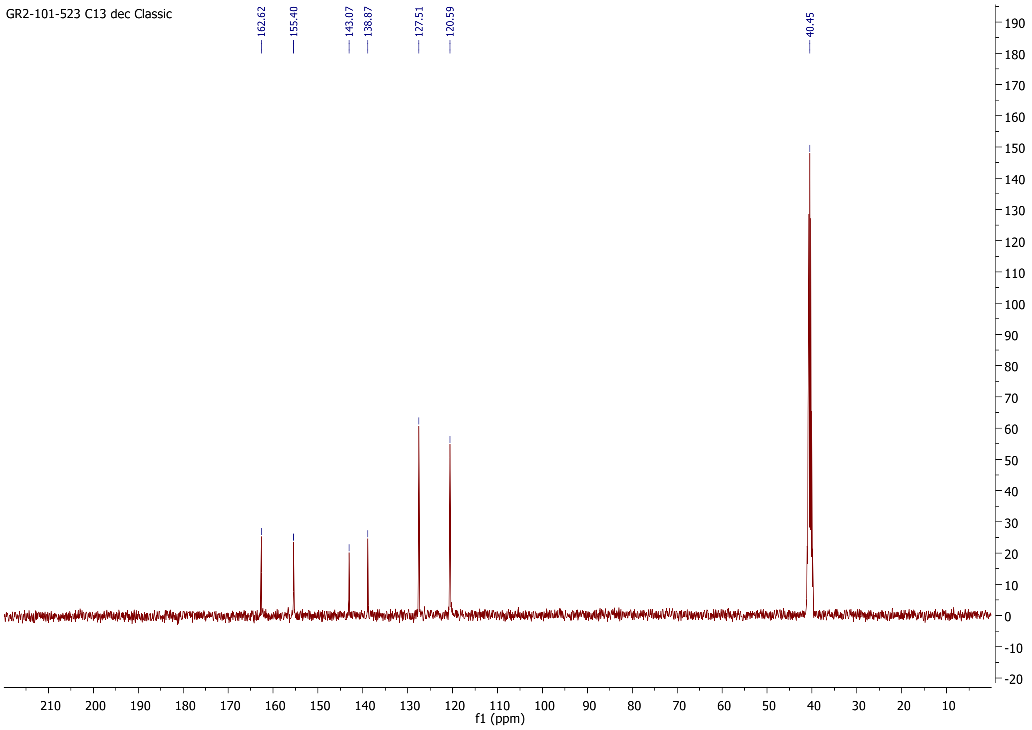

^{1}H NMR spectrum of compound **5b** (400 MHz, DMSO-*d6*)

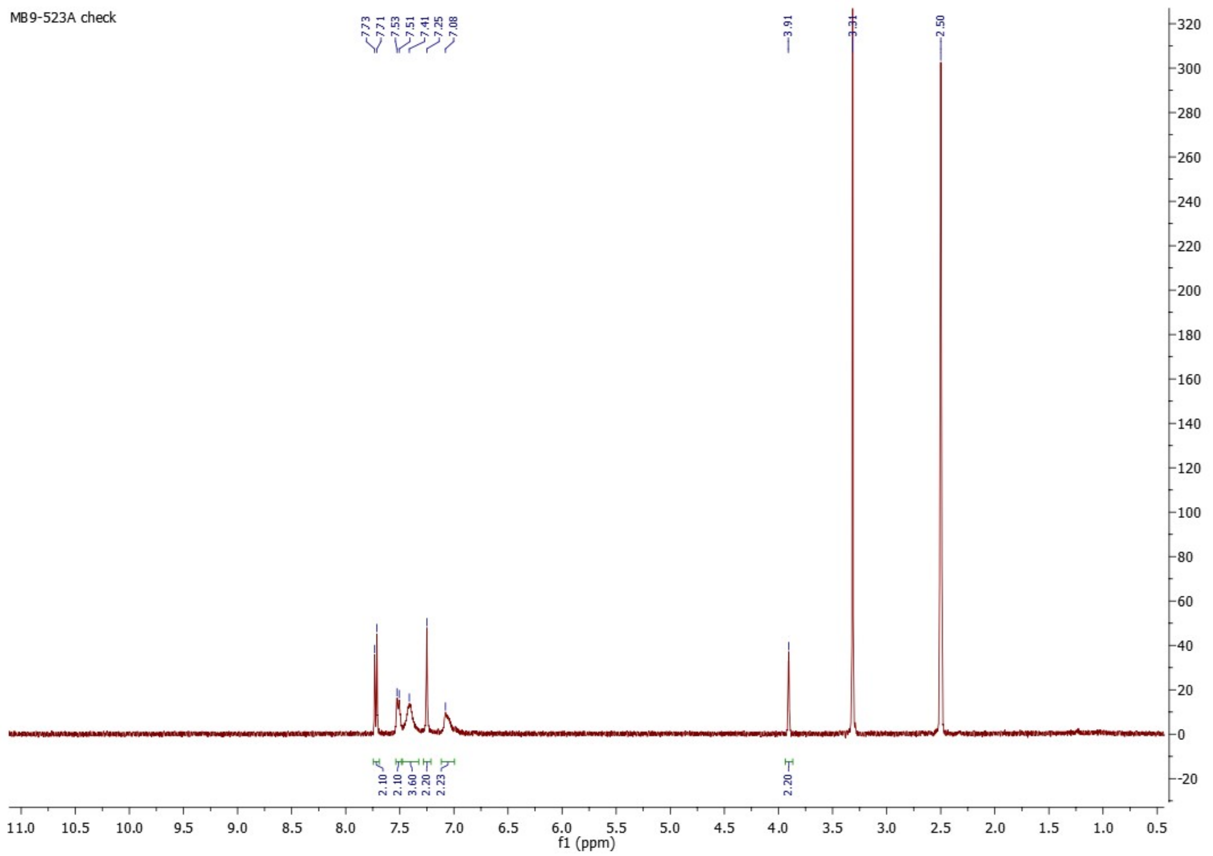

^{13}C NMR spectrum of compound **5b** (100 MHz, DMSO-*d6*)

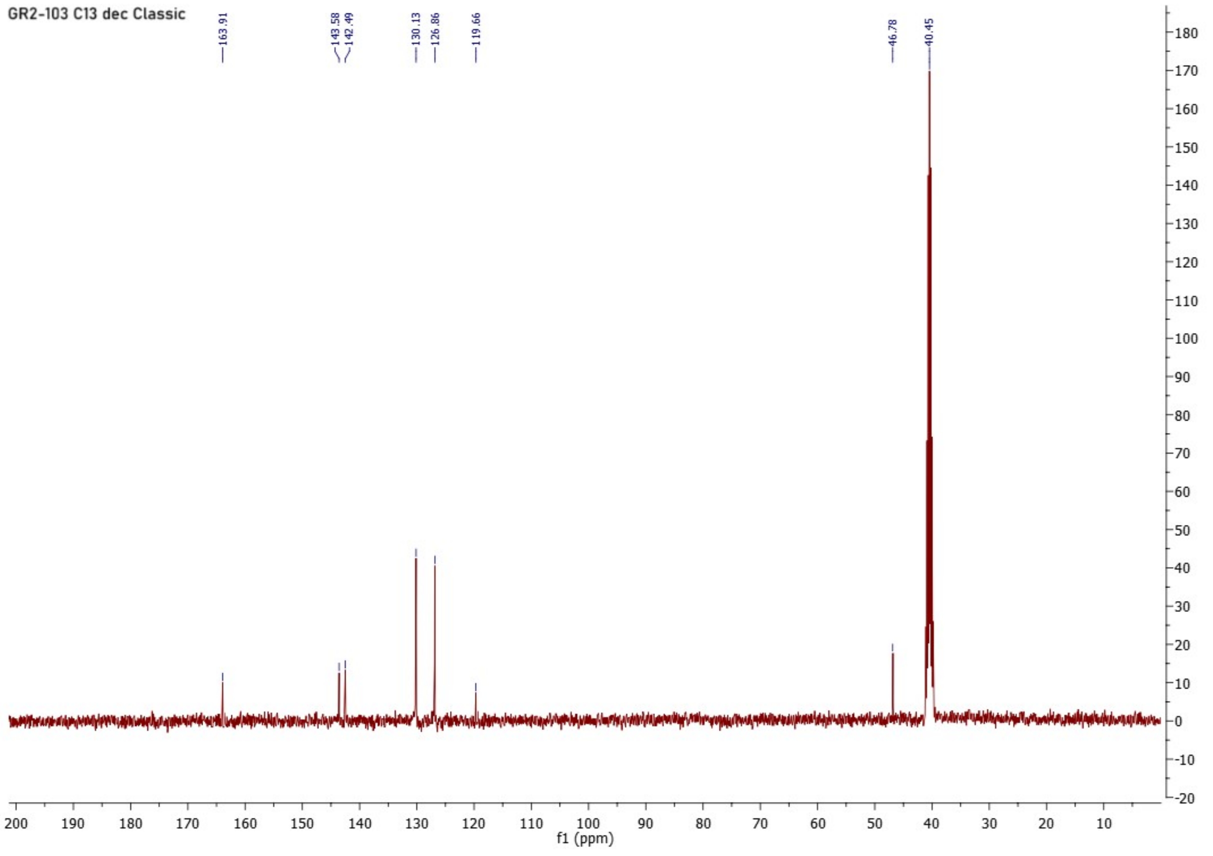

^{1}H NMR spectrum of compound **5c** (400 MHz, DMSO-*d6*)

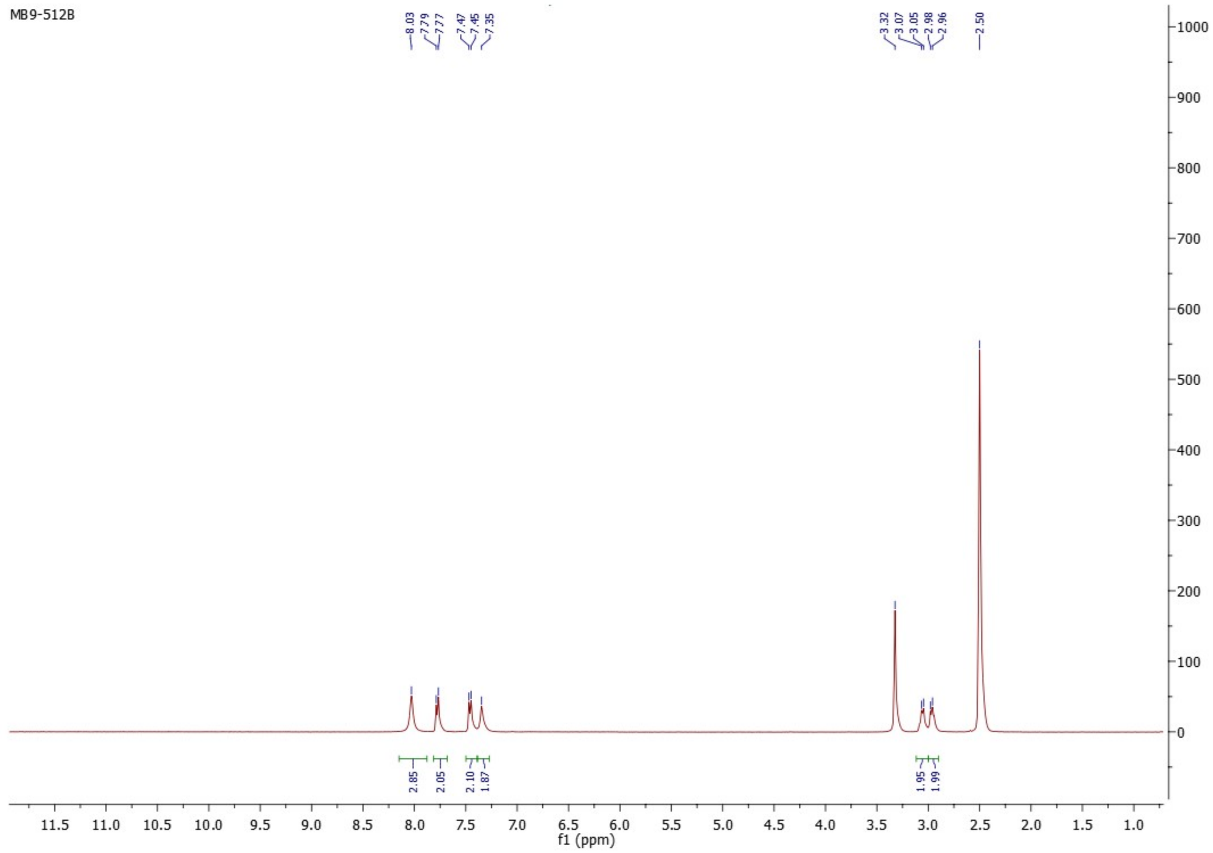

^{13}C NMR spectrum of compound **5c** (100 MHz, DMSO-*d6*)

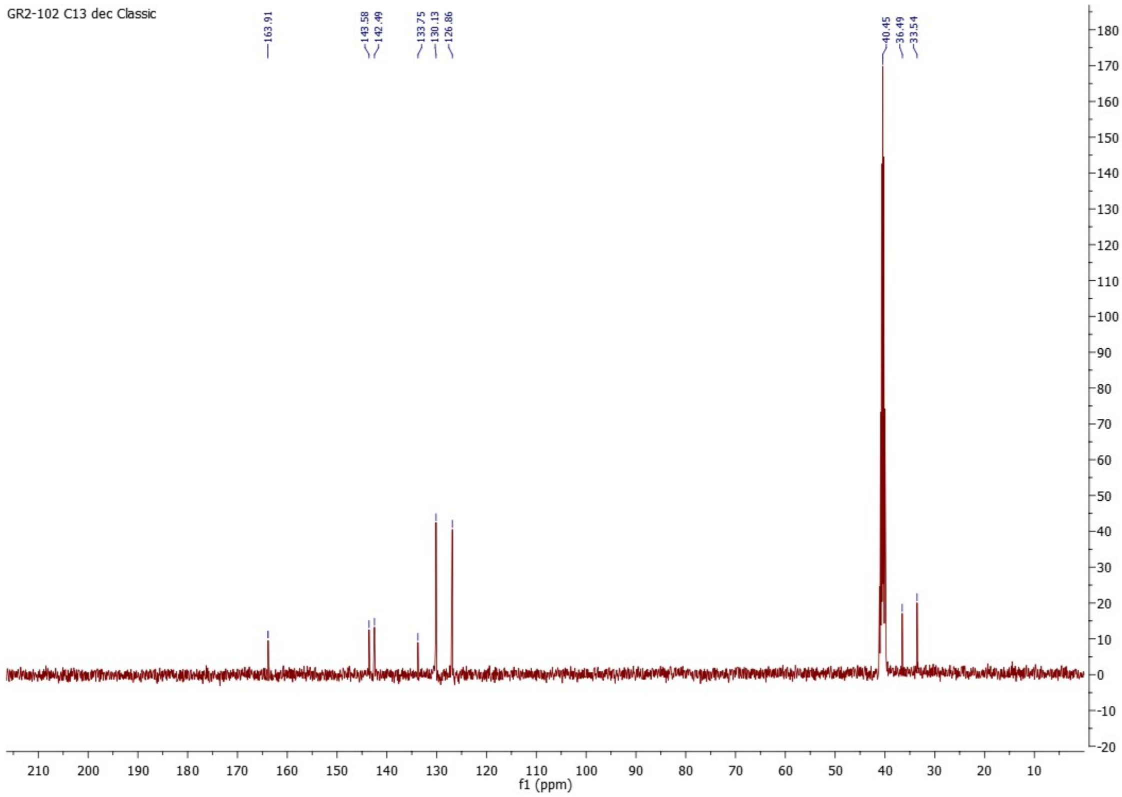

Table S1. Summary of Data Collection and Refinement Statistics Values in brackets refer to the highest resolution shell.

Data Collection statistics			
	**CA II-5b**	**CAII-5b with glycerol**	**CA XII-5b**
PDB ID	8ROU	8ROW	8RNS
Wavelength (Å)	1.0001	0.9718	1.0001
Space Group	P 2_1	P2_1	P 21
Unit cell (a, b, c, α, β, γ) (Å,)	42.28 41.09 72.29 90 104.20 90	42.44 41.30 72.03 90 104.29 90	41.98 41.33 71.81 90 103.87 90
Limiting resolution (Å)	70.08-1.08 (1.10-1.08)	40.02-1.05 (1.07-1.05)	40.76-1.14 (1.16-1.14)
Unique reflections	102568 (5042)	107024 (4661)	85727 (3897)
Rmerge	0.081 (1.403)	0.049 (0.706)	0.07 (1.351)
Rmeas	0.089 (1.591)	0.054 (0.801)	0.076 (1.547)
Redundancy	5.9 (4.5)	6.1 (4.5)	5.9 (4.3)
Completeness overall (%)	99.8 (99.7)	95.0 (83.7)	98.2 (90.0)
<I/σ(I)>	9.8 (1.1)	16.7 (2.2)	12.0 (1.1)
CC (1/2)	1.0 (0.3)	1.0 (0.7)	1.0 (0.4)
**Refinement statistics**			
Resolution range (Å)	70.08-1.08	40.02-1.05	40.76-1.14
Rfactor	0.1301	0.1160	0.1415
Rfree	0.1515	0.1386	0.1614
r.m.s.d. bonds (Å)	0.0118	0.0124	0.0113
r.m.s.d. angles ( )	1.8997	1.9011	1.7661
**Ramachandran statistics (%)**			
Most favored	97.3	97.3	96.9
additionally allowed	2.7	2.7	3.1
Outlier regions	0.0	0.0	0.0
**Average B factor (Å^2)**			
All atoms	15.1	13.9	17.9
Inhibitors	14.6	13.6 / 23.1	23.5
Solvent	17.1	15.8	20.0

Table S2. Summary of Data Collection and Refinement Statistics Values in brackets refer to the highest resolution shell.

Data Collection statistics	
	**CA II-5c**
PDB ID	9H0V
Wavelength (Å)	1.0002
Space Group	P 21
Unit cell (a, b, c, α, β, γ) (Å,)	42.43 41.26 72.47 90 104.72 90
Limiting resolution (Å)	35.56-1.40 (1.42-1.40)
Unique reflections	43258 (2316)
Rmerge	0.040 (0.200)
Rmeas	0.044 (0.216)
Redundancy	6.2 (6.7)
Completeness overall (%)	90.3 (97.4)
<I/σ(I)>	24.2 (8.6)
CC (1/2)	0.999 (0.981)
	**Refinement statistics**
Resolution range (Å)	35.56-1.40
Rfactor	0.0991
Rfree	0.1380
r.m.s.d. bonds (Å)	0.0105
r.m.s.d. angles ( )	1.9264
	**Ramachandran statistics (%)**
Most favored	96.1
additionally allowed	3.9
Outlier regions	0.0
	**Average B factor (Å^2)**
All atoms	16.8
Inhibitors	22.4 / 30.9
solvent	31.6

>hCA II

MSHHWGYGKHNGPEHWHKDFPIAKGERQSPVDIDTHTAKYDPSLKPLSVSYDQATSLRILNNGHAFNVEFDDSQDKAVLKGGPLDGTYR
LIQFHFHWGSLDGQGSEHTVDKKKYAAELHLVHWNTKYGDFGKAVQQPDGLAVLGIFLKVGSAKPGLQKVVDVLDSIKTKGKSADFTNF
DPRGLLPESLDYWTYPGSLTTPPLLECVTWIVLKEPISVSSEQVLKFRKLNFNGEGEPEELMVDNWRPAQPLKNRQIKASFK

>hCA XII mimic

MSHHWGYGKHNGPEHWHKDFPIAKGERQSPVDIDTHTAKYDPSLKPLSVSYDQATSLRILNNGH**S**F**K**V**N**FDDSQDKAVLKGGPLDGTYR
L**T**QFHFHWGSLDGQGSEHTVDKKKYAAELHLVHWNTKYGD**AS**KA**S**QQPDGLAVLGIFLKVGSAKPGLQKVVDVLDSIKTKGKSADFTNF
DPRGLLPESLDYWTYPGSLTTPPL**N**E**T**VTWIVLKEPISVSSEQVLKFRKLNFNGEGEPEELMVDNWRPAQPLKNRQIKASFK

hCA II	MSHHWGYGKHNGPEHWHKDFPIAKGERQSPVDIDTHTAKYDPSLKPLSVSYDQATSLRIL	60
hCA XII	MSHHWGYGKHNGPEHWHKDFPIAKGERQSPVDIDTHTAKYDPSLKPLSVSYDQATSLRIL	60
	NNGH**A**F**N**V**E**FDDSQDKAVLKGGPLDGTYRL**I**QFHFHWGSLDGQGSEHTVDKKKYAAELHL	120
	NNGH**S**F**K**V**N**FDDSQDKAVLKGGPLDGTYRL**T**QFHFHWGSLDGQGSEHTVDKKKYAAELHL	120
	VHWNTKYGD**FG**KA**V**QQPDGLAVLGIFLKVGSAKPGLQKVVDVLDSIKTKGKSADFTNFDP	180
	VHWNTKYGD**AS**KA**S**QQPDGLAVLGIFLKVGSAKPGLQKVVDVLDSIKTKGKSADFTNFDP	180
	RGLLPESLDYWTYPGSLTTPPL**L**E**C**VTWIVLKEPISVSSEQVLKFRKLNFNGEGEPEELM	240
	RGLLPESLDYWTYPGSLTTPPL**N**E**T**VTWIVLKEPISVSSEQVLKFRKLNFNGEGEPEELM	240
	VDNWRPAQPLKNRQIKASFK	260
	VDNWRPAQPLKNRQIKASFK	260

Figure S1. Amino acid sequence alignment of hCA II and hCA XII. Residues of hCA II bolded in red were replaced with the corresponding residues from hCA XII to create the mimic proteins.
